# Action Identity in Style Simulation Systems: Do Players Consider Machine-Generated Music As of Their Own Style?

**DOI:** 10.3389/fpsyg.2016.00474

**Published:** 2016-05-06

**Authors:** Armen Khatchatourov, François Pachet, Victoria Rowe

**Affiliations:** ^1^Sony Computer Science Laboratory ParisParis, France; ^2^College of Social Science and International Studies, University of ExeterExeter, UK

**Keywords:** self-recognition, action identity, style, machine-learning, artificial intelligence, Markov models

## Abstract

The generation of musical material in a given style has been the subject of many studies with the increased sophistication of artificial intelligence models of musical style. In this paper we address a question of primary importance for artificial intelligence and music psychology: can such systems generate music that users indeed consider as corresponding to their own style? We address this question through an experiment involving both performance and recognition tasks with musically naïve school-age children. We asked 56 children to perform a free-form improvisation from which two kinds of music excerpt were created. One was a mere recording of original performances. The other was created by a software program designed to simulate the participants' style, based on their original performances. Two hours after the performance task, the children completed the recognition task in two conditions, one with the original excerpts and one with machine-generated music. Results indicate that the success rate is practically equivalent in two conditions: children tended to make correct attribution of the excerpts to themselves or to others, whether the music was human-produced or machine-generated (mean accuracy = 0.75 and = 0.71, respectively). We discuss this equivalence in accuracy for machine-generated and human produced music in the light of the literature on memory effects and action identity which addresses the recognition of one's own production.

## Introduction

Recent progress in artificial intelligence and statistical inference make it possible to generate artificially musical material in a given musical style. From the viewpoint of artificial intelligence, a style is essentially a statistical object, characterized by the statistics of occurrence of the various elements making up a musical production (e.g., notes), as well as the statistics of their inter-relationships. The increasing sophistication of style-based music generation systems raises novel questions at the frontier of artificial intelligence and the psychology of music perception. Among those, an important question is to what extent the human player considers the musical result of this generation as reproducing his or her own style.

Musical style simulation has been the subject of many studies in artificial intelligence resulting in a steady stream of software attempting to imitate musical style (see e.g., Thomas et al., 2013). Most of these systems are based on Markov models of music: they exploit statistics concerning the temporal succession of musical events (e.g., the frequency of event *Do* followed by the event *Re* in a given corpus), and reapply these statistics for generating new music material (see Section Style Simulation Software, below). For the experiment described in this paper we used the MIROR-IMPRO system, itself based on a system known as Continuator (Pachet, 2003; Addessi and Pachet, 2005). MIROR-IMPRO recombines the user's musical input according to a Markovian generation scheme, to create new musical phrases which are played back to the user.

Do subjects consider as their own style music that has been machine-generated by recombining their original material so as to maintain stylistic consistency? Do the machine-generated excerpts sound like “my music?” We address this research question by comparing the accuracy of style attribution of machine-generated musical excerpts with the accuracy of self-recognition task in human-produced excerpts.

Style attribution accuracy can be measured as the success rate in identification of musical excerpts as “sounding like my music” (see Section Experiment on Style Attribution for details).

We focus on free-form improvisations performed by musically naïve school-age children, because this form of musical expression does not assume any prior musical knowledge (see Section Human Performance in Recognition of Action Identity, below). Here, possible self-attribution takes precedence over musical genre and sole mastery of the instrument.

The rest of the paper is organized as follows. The first section covers the state of the art on the recognition of one's own actions in musical domain. The second section describes the main principles underlying the computer software used in our experiment. The third section presents the experiment which compares attribution accuracy for human-produced and machine-generated excerpts. The fourth section discusses which cognitive mechanisms may underpin self-attribution, provides some insights for further analysis of this question, and discusses the role of musical structure in style attribution.

## Human performance in recognition of action identity

The question of recognizing the products of one's own action has been addressed in a systematic manner by Knoblich and Flach (2003) who introduced the term “action identity” to describe the recognition of “one's own way of doing things, one's action style.” In accordance with this approach, we define the style through style-attribution: if I consider actions as my own, I recognize my “action style.”

How good are humans at differentiating between the products of their own actions and those of others, in creative domains such as drawings, handwriting or the musical phrases they have performed or improvised? What factors may influence the recognition of one's action identity (Knoblich and Flach, 2003)?

Action-perception mechanisms of recognition are grounded in the establishment of the appropriate links between the actions and their perceptual consequences, as emphasized by the ecological theory of perception (Gibson, 1979). Recent approaches such as the common coding theory (Prinz, 2002), action simulation theories (Jeannerod, 2001; Dokic and Proust, 2002), and the “simulation” theory of cognitive function (Hesslow, 2002), put forward the argument that the observer's action system is most strongly activated during the perception of self-produced actions. In other words, a closer match between anticipated actions and perceived effect leads to this self-recognition capacity. Note that “(internal) action *simulation*” refers to an explanatory mechanism postulated to exist in humans, whereas “style *simulation* software” refers to particular software imitating the style of the player (see below).

Most experimental research in this framework has dealt with adults. For instance, in the study of Flach et al. (2004) non-trained participants were asked to recognize recordings of their own clapping patterns among recordings of other participants' clapping. This experiment provided a measure of the individuals' discrimination capability and their response bias (see Discussion section for details on these measures). The participants were able to identify their own production under two test conditions: original recordings of clapping and altered recordings (tone sequences reproducing the temporal succession of the maximum amplitudes of the claps). On the basis of these findings, the authors argue that the general temporal pattern plays an important role in self-recognition.

Repp and Knoblich (2004) reported a study in which trained pianists were asked to record unfamiliar classical pieces and then to identify their own performances from amongst those of other players. The experiment measured self and other recognition on a scale from one to five, and confirmed that the participants were able to identify their own production both at the main test 2 months after the recording and during a follow-up test two more months later. The follow–up test was performed with altered musical material in two conditions: retaining information about articulation, timing, and dynamics (intensity or velocity) of the originally performed melody; and retaining articulation and timing only. No significant difference was found between the two follow-up conditions. To summarize, expressive timing and articulation were sufficient to maintain self-recognition capacity, while tempo and overall dynamic level did not play an important role.

These results provide important insights. First, adult players clearly exhibit the ability to distinguish between their own way of playing and someone else's. This is true both for a short time after the production phase and up to several months later. Secondly, the literature suggests that the ability to recognize one's own production can withstand alterations of musical dimensions such as intensity, pitch or tempo. By enforcing changes in these dimensions, the question was to what extent recognition may be robust to degradations. In the present study we are interested in exploring stylistic variations which are machine-generated material explicitly built to enforce stylistic properties. That is, we relate the simulation of a person's style to the accuracy of the style attribution.

To the best of our knowledge, the question of recognition of the action style has not yet been addressed in children. Several studies showed that children as young as 6 years old are able to successfully classify musical excerpts according to the genre criteria (Gardner, 1973; Marshall and Shibazaki, 2011). Moreover, it has been shown that children are able to associate emotional judgments with musical elements such as change from major to minor (Dalla Bella et al., 2001), and that they are able to exhibit sophisticated musical discrimination in areas such as “melodic perception, phrase learning, melodic reproduction with varying harmonies and timbres, and rhythmic ability” (Zimmerman, 1971). Based on these findings, school-age children can be assumed to possess the necessary cognitive abilities for recognition of action identity.

## Style simulation software

The notion of musical style has been vigorously addressed in the community of Artificial Intelligence and Computer Music. Studies by Cope (1991), Ebcioğlu (1990), or Steedman (1984) have demonstrated that computers could produce original musical works that sounded like the works of existing composers, at least concerning specific dimensions of the music. Though many methods have been explored to model style, the most practical and successful ones are based on the modeling of style as a statistical object (Nierhaus, 2009). Those methods lead naturally to systems that can capture style in real-time from examples performed by a user, as opposed to methods involving the manual coding of a set of rules, such as the three studies mentioned above. These methods have gained popularity in the recent years, and are used in a variety of contexts (see e.g., Thomas et al., 2013).

Statistical methods are mostly based on Markov chains or variants thereof. Markov chains are statistical models whose relative simplicity is due to the underlying Markov hypothesis, which states that the future state of a sequence depends only on the current state. Markov chains are defined as the set of probabilities for any event (e.g., note) to succeed to any other. For instance, a Markov chain captures the probability that a note, say *Do* is followed by note *Re* in the user's corpus. Many attempts to use Markov chains to model musical style have been made, including refinements to the Markov hypothesis, such as variable contexts lengths for defining transition probabilities. However, non-local properties of musical style are difficult to model with Markov-based approaches, because they essentially violate the Markov hypothesis. New methods for capturing and generating Markov chains have been introduced to address this problem (Pachet and Roy, 2011). These methods, based on combinatorial optimization as opposed to random walks, propose cost efficient solutions to generate Markov sequences that satisfy various constraints such as melodies that start or end by specific notes (Pachet et al., 2011). The MIROR-IMPRO software used in the experiment reported here is based on these techniques.

From the user's point of view, the MIROR-IMPRO software can generate different types of output melodies based on the user's musical input. Initially, the user plays / improvises on a MIDI keyboard connected to the software. The melodies played constitute a *training set*, associated to the user and saved. This training set is later restored to build a corpus of pieces using the constrained Markov techniques described above. Thus, the machine-generated output is composed of what the user might have played, i.e., a constrained recombination of musical elements previously played by the user. If the user still self-attributes that machine-generated music, the “style” was then successfully simulated.

## Experiment on style attribution

In the experiment described below, we compare the accuracy of style attribution of machine-generated musical excerpts with the accuracy of self-recognition in human-produced music. We expect the attribution accuracy for machine-generated music to be practically equivalent to the baseline set by the self-recognition accuracy for human-produced music.

### Materials and methods

#### Participants

Fifty-six school-age children (25 males and 31 females) participated in the study. They were all between 7 and 10 years old and came from 3 schools in the UK. None of the participants had received any intensive musical training before the experiment. The 56 participants were divided into 14 groups of four participants. Note that the division in groups was only for the purpose of constituting the training set (as explained below) and the participants were not aware of it. This study was carried out in accordance with the recommendations of Exeter University ethical guidelines and the Declaration of Helsinki, with written informed consent from all subjects' parents.

#### Materials

In the *Human-produced* condition, the musical excerpts (also called melodies) to be recognized by the participant are exact recordings of what has been played in the training set phase (which is explained below), including ones the participant has previously improvised and those improvised by others (and that the participant has not heard before). The excerpts longer than 10 s are cut, so that the length cannot be used as a recognition criterion. This condition gives a baseline for the participants' capacity to recognize their own style.

In the *Machine-generated* condition, the excerpts are machine generated pieces, including pieces composed from the material the participant has played before, as well as pieces composed from the musical material of other participants in the group. A specific procedure is used to generate machine composed excerpts, which are generated as the sequence of 4 chunks of 4 events (i.e., notes or chords) each. The sequence of chunks defines a high-level musical structure of the piece and is generated as follows: statement, continuation, continuation, and conclusion. A “statement” begins with one of the starting notes from the training set, and produces a phrase ending on a random pitch. A “continuation” continues from the last note of the previous chunk, and its terminal note will be the terminal note of one of the sequences in the training set. A “conclusion” continues from the previous chunk and will end on the note which was the first note of the piece (i.e., the first note of the first chunk). All the intermediary notes in the four chunks will obey the principles of Markovian generation as described above, with an additional constraint of not being starting or ending notes (i.e., notes that began or ended any of the input improvisations in the training set).

### Design and procedure

#### Training set phase

Prior to the test phase, each participant was asked to sit at a table where a Korg X50 music synthesizer was connected to a laptop with the MIROR-IMPRO software. Then, each child was asked to “play as he/she wished,” i.e., to perform free-form improvisation, with no restriction or guidance concerning the nature of the phrases to play. There was only one child at a time in the room. The experimenter would only indicate to the participant to stop playing when enough melodies were played for the system to generate the pieces. This number was defined in a prior pilot experiment and was about 100 musical events (notes or chords) per participant. This musical material was recorded in groups of 4 children (who were unaware of the division in groups), and filtered to meet a minimum length of 4 events (notes or chords) for each melody played. In the test phase described below, the participants in a given group were exposed only to their own material or to the material from their group mates, either in the form of exact recordings, or in the form of machine-generated pieces based on the training set of this particular group. This recording procedure was repeated for 14 groups.

#### Test phase

The test phase took place 2 h later on the same day and the same material set-up was used. During the intervening time, the children pursued normal school activities with no relation to musical subjects. The experiment used a within-subjects design, with all the participants being exposed to the two conditions. The within-subjects variable was the nature of the musical excerpt to be recognized, i.e., *Human-produced* (an extract from original performance) or *Machine-generated*. The dependent variable was the accuracy of the recognition.

The participants were presented with 16 excerpts: eight excerpts in *Human-produced* condition, randomized between self-related and other-related excerpts, and then eight excerpts in *Machine-generated* condition randomized between self-related and other-related excerpts, both conditions being within the same block with no delay between them.

They had to answer *yes* or *no* to the question “Does this sound like your music?.” No indications were given prior to listening and no feedback was provided to the participants as to the nature of the musical excerpt they had listened to; the experimenter was unaware of the correct answers at the time of the test. The answer was recorded as one if correct and zero if incorrect. The correct answer in *Human-produced* condition is when the excerpt that the participant had played prior to the test was correctly identified as “mine,” or if another player's excerpt was correctly rejected as “not mine.” The correct answer in *Machine-produced* condition is when the excerpt that was generated from the participant's training set was correctly identified as “mine,” or when the excerpt based on other's material was correctly rejected as “not mine.” In both conditions, the chance level accuracy is 0.5, that is the half of excerpts are self-related and the other half of the excerpts are other-related.

The procedure was then repeated for each of the four participants in the group, and then for 13 more groups. The experimental data obtained was paired and stratified by group. The total number of observations was *N* = (8 + 8) × 4 × 14 = 896.

### Statistical treatment and data

#### Statistical methods

We use a combined statistical approach, performing both the classical “Null Hypothesis Significance Test” (NHST) and “Equivalence Hypothesis Testing” (EHT). The latter approach comes from bioequivalence studies and aims to evaluate whether the test condition is acceptably equivalent to the reference condition. From the practical point of view, the EHT can be conducted either as the two one-sided tests procedure (TOST) or as the confidence interval (CI) analysis.

In TOST procedures, the null hypothesis is that the mean responses of the two conditions are different, and the alternative hypothesis is that the mean responses are equivalent within some small range *delta* defined by the experimenter. The *p*-value is then associated with the probability of Type I error, that is, to conclude that the treatments are equivalent when in fact they are not. The two treatments are declared equivalent within the range *delta* when the null hypothesis is rejected. In CI procedures, the basic approach is to compute a statistical confidence interval around the sample mean differences and to determine if it lies within the equivalence region previously defined. As both methods have been shown to be equivalent (Chow and Liu, 1992; Tango, 1998), we report the equivalence confidence intervals only.

The main practical issue with the EHT approach is to choose an adequate *delta* value, which is usually done on the basis of common standards and previous knowledge of the domain of interest. In domains other than bioequivalence (including but not limited to the software engineering), different delta levels have been explored (Dolado et al., 2013), including different percentages (ranging from 10 to 50%) of the standard deviation of the differences. In our case, we use the standard delta equal to 20% of the lowest value of the mean of the reference variable (that is 20% of the success rate in the *Human-produced* condition, for the original dataset obtained in the experiment and called *a1*). Transposed onto the scale of accuracy (or success rate) in the recognition task the delta is then equal to 0.15.

As the data obtained does not fully satisfy the criteria of normality, we have chosen to use non-parametric methods for the analysis, and in particular Hodges-Lehmann estimate based on rank procedures for CI.

#### Data homogeneity

Before proceeding to the statistical treatment, it is of interest to determine whether there is a relationship between the condition and the accuracy, adjusted for groups. No heterogeneity of stratum specific odds ratio (OR) was found (Woolf test X-squared = 11.89, *df* = 13, *p* = 0.54). A Mantel–Haenszel exact procedure was then performed to provide a pooled OR of 0.77 [0.57–1.05], *p* = 0.09. That is, there is no significant evidence rejecting the null hypothesis of homogeneity. Overall, the data do not suggest an association between condition and accuracy when adjusted for group. It is then acceptable to test the overall pattern of difference between *Human-produced* and *Machine-generated* conditions on the pooled data.

## Results

### General results

**Figure 1:**
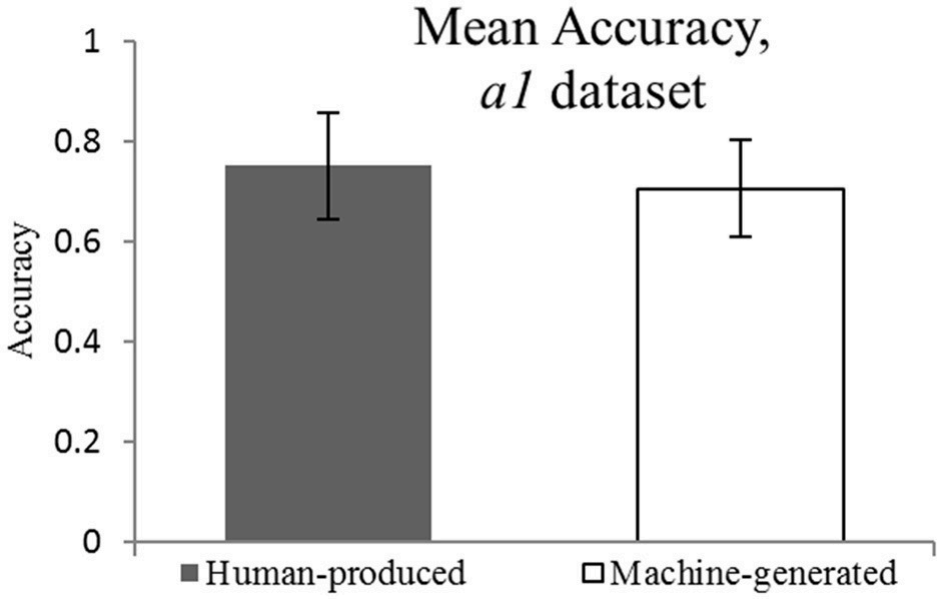
**Mean Accuracy for *a1* dataset**. *M* = 0.75 and *M* = 0.71.

**Figure 2:**
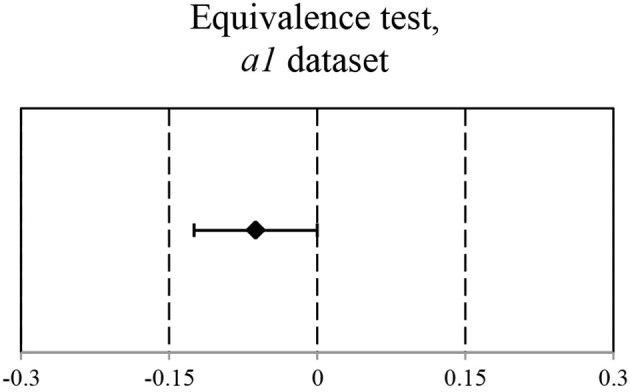
**Ninety percent CI for Mean(Machine-generated) – Mean(Human-produced): (−1,25e–1; 2,96e–5)**. CI is within the equivalence interval of (−0.15; 0.15).

The original data set *a1* (see Figure 1) suggests that there is no significant difference between the accuracy of self-recognition for the melodies played by participants and the accuracy of style attribution for the machine-generated pieces (*M* = 0.75, *M* = 0.71, respectively, against the chance level of 0.5). The EHT results on the *a1* dataset show that the equivalence within the specified equivalence bound of 0.15 can be claimed. These findings are reflected in Figure 2.

**Figure 3:**
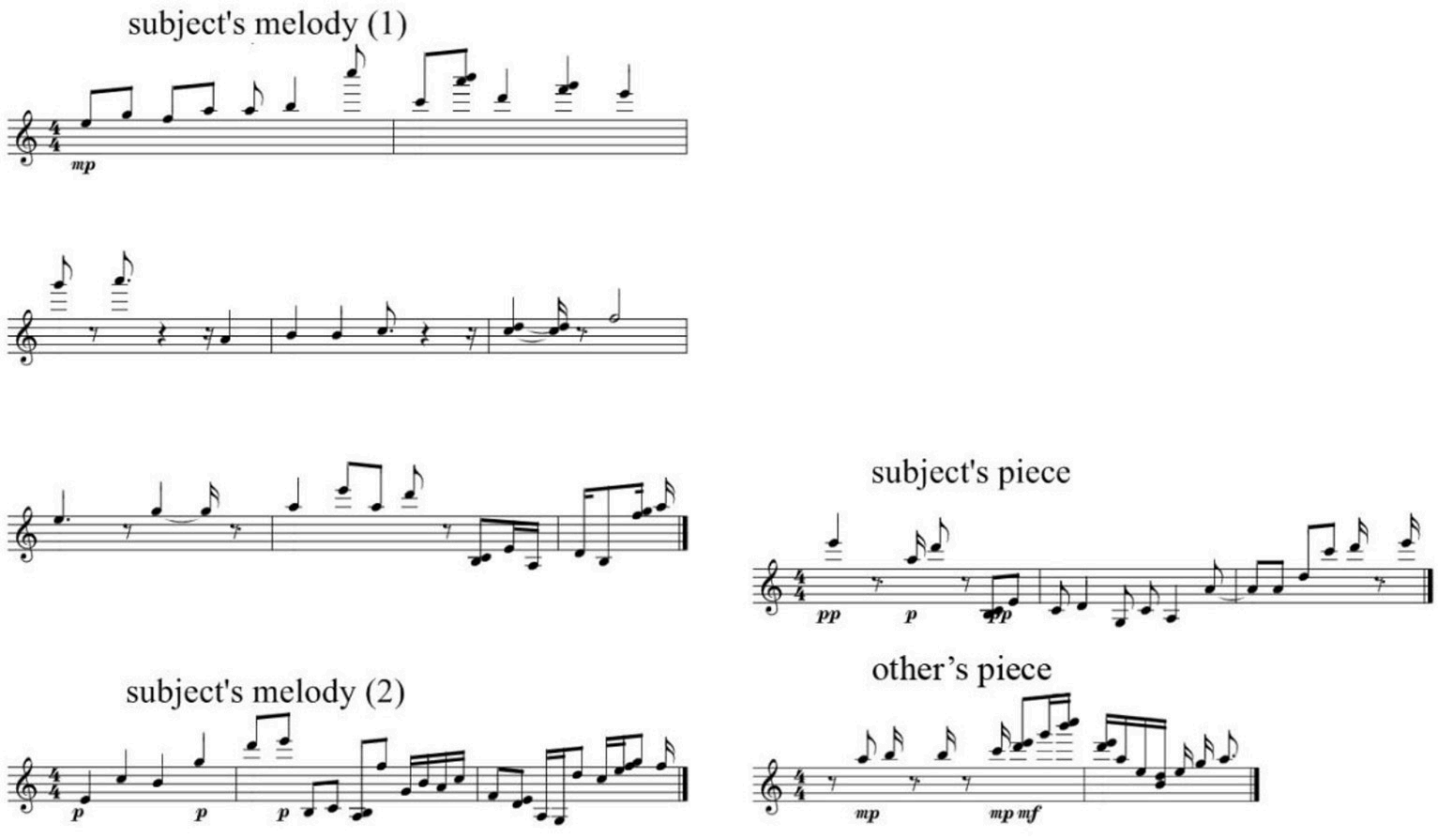
**Participant's input (left) and a successful attribution of one's own style in the machine-generated piece (top right), as well as a correct rejection of a machine-generated piece based on other user's musical material (bottom right)**. See in Supplementary Material, Audio 1 for Subject's melody 1, Audio 3 for Subject's melody 2, Audio 2 for Subject's piece, Audio 4 for Other's piece.

Participants were able to distinguish pieces based on their own material as successfully as they did for recorded melodies, even when this recognition was not obvious. Figure 3 gives an example of such a successful attribution of one's own style (illustrated by two melodies played by the participant), while correctly rejecting pieces based on other user's material.

### Results refined to measure the “true” accuracy

In the experiment described above, the very purpose of the discrimination task leads to a juxtaposition of the user's material and the material from other participants. The effects of this juxtaposition are discussed below.

**Figure 4:**
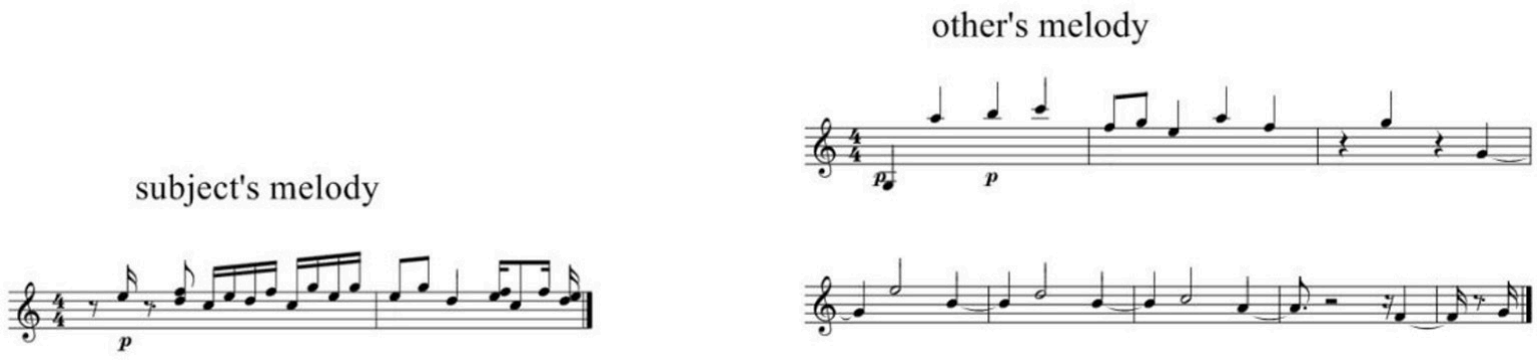
**False positive response in *Human-produced* condition, due to the stylistic similarity between two participants**. Participant's input during training phase **(left)** and other user's melody erroneously rated by the participant as “my style” **(right)**. See in Supplementary Material, Audio 5 for Subject's melody, Audio 6 for Other's melody.

**Figure 5:**
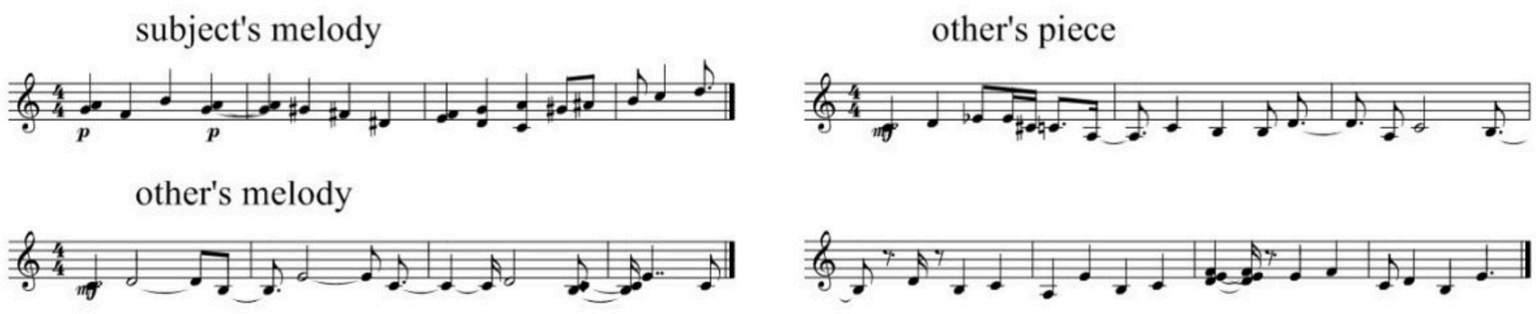
**False positive response in Machine-generated condition, due to the stylistic similarity between two participants**. Participant's input during training phase **(top left)**, input of another user from the same group during training phase **(bottom left)**, and a machine-generated piece erroneously rated by the participant as “my style” **(right)**. See in Supplementary Material, Audio 7 for Subject's melody, Audio 8 for Other's melody, Audio 9 for Other's piece.

In our experimental situation, unintended stylistic similarity between participants in the group can create a specific explicable type of attribution errors present in both *Human-produced* and *Machine-generated* experimental conditions. A false positive response can occur if another participant in the group has played with a style similar to the tested participant. The former case (*Human-produced*), illustrated in Figure 4, makes it impossible to distinguish “my” melody from the “other's” melody. The latter case (*Machine-generated*), illustrated in Figure 5, makes it impossible to distinguish “my” machine-generated piece from the “other's” piece.

Revealing these false positive responses and adjusting for them can give a measure of the “true” accuracy: it might happen that revealing these errors causes the “true” accuracy for machine-generated pieces to be lower than the “true” accuracy for human-produced melodies. If this were the case, our previous conclusion that individual musical style had been successfully preserved might be erroneous. It is therefore important to answer the following question: Is there an influence of these specific errors on the difference between accuracy for melodies and for pieces?

To address this issue, we performed a qualitative analysis of the data by listening to all the cases where a discrimination error was present. As for these “explainable” attribution errors, there were 11 occurrences spread among nine participants for the *Human-produced* condition, and eight occurrences spread among eight participants for the *Machine-generated* condition. The data set *a2* was obtained from the data set *a1* by dropping the corresponding observations.

**Figure 6:**
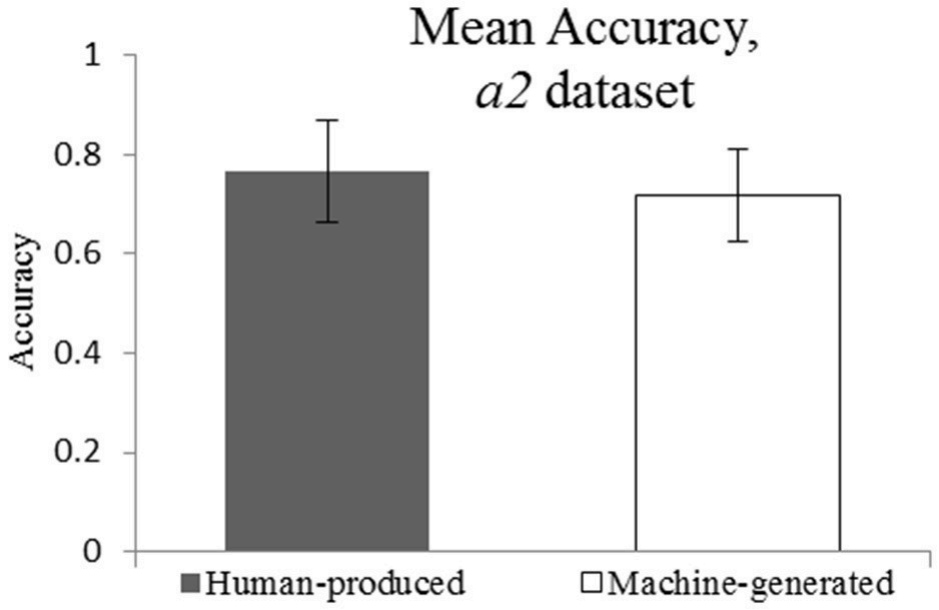
**Mean Accuracy for *a2* dataset (explainable errors dropped)**. *M* = 0.77 and *M* = 0.72.

**Figure 7:**
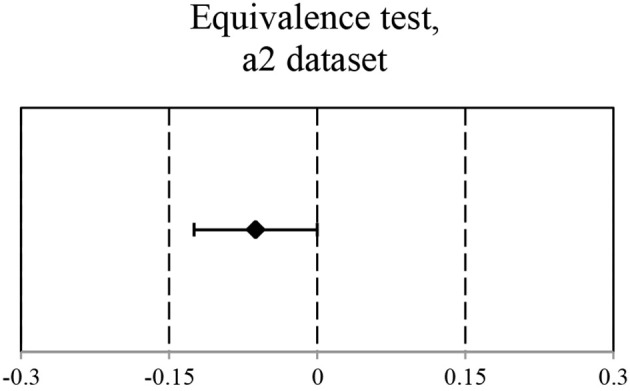
**Ninety percent CI for Mean(Machine-generated) – Mean(Human-produced): (−1,25e–1; 3,45e–5)**. CI is within the equivalence interval of (−0.15; 0.15).

The adjusted data set *a2* (see Figure 6) suggests that dropping the “explainable” attribution errors does not change the conclusion about the style attribution accuracy. Indeed, the mean accuracy for *Human-produced* and *Machine-generated* conditions was of 0.77 and 0.72, respectively. The EHT results show that the equivalence within the specified equivalence bound of 0.15 can be claimed. These findings are reflected in Figure 7.

Overall, the experiment allows us to conclude that the participants were able to recognize their own style among human-produced and machine-generated excerpts, and that the accuracy of style attribution for machine-generated excerpts is practically equivalent to the accuracy of self-recognition in human-produced excerpts.

## Discussion on human performance in recognition of action identity

In this experiment, we observed that musically naïve school-age children identify machine-generated music as sounding like “their own” music if it was generated from their own previous production. This result indicates that the MIROR-IMPRO software used in this experiment was able to simulate the participant's style. What are the factors that influence the style attribution of machine-generated excerpts? More specifically, we discuss the role of memory and the impact of high-level structural musical aspects on style attribution, and we propose avenues of further research.

### Episodic memory

It could be argued that episodic memory might have played a dominant role in the style attribution capacity. However, there are at least two arguments which suggest that factors other than pure memory are in action here. The first argument concerns action representations in general. As the studies already cited (Flach et al., 2004; Repp and Knoblich, 2004) show, it is difficult to make a clear-cut distinction between memory effects and the recognition of the self-generated musical productions. This is due to the fact that both episodic memory and action representations are acquired by experience. It is likely however that knowledge of one's own production is not episodic but generative in nature: this knowledge does not presuppose a conscious recollection of the episode but an internal simulation of the action and its perceptual consequences. Moreover, in domains other than musical perception, there is some evidence of a close link between ownership and memory advantage. For example, Cunningham et al. (2008) show that in the visual field even transient self-ownership of items improves their memorability. Therefore, in this framework the question is not whether self-attribution rests on the memory processing, but if the memory processing at stake during self-attribution is of a different nature.

The second argument concerns our particular experiment. A child's recognition of machine composed pieces as “being in my style” cannot be explained by memory effects only: the participants neither heard the pieces before the test phase, nor were they aware of their machine-composed nature. In other words, a conscious recollection of the episode of the action is, strictly speaking, not at stake in our case.

Therefore, style attribution cannot be accounted for by episodic memory effects only, and the recognition of self-related items is different from episodic memory of “neutral” events. It can be concluded that participants “remembered” not the excerpts themselves but the way they played, i.e., their own style, and correctly attributed them when the machine-generated pieces kept indeed elements of this individual style.

### Influence of high-level musical structure on style attribution

As has been highlighted in the sub-section Materials, the machine-generated pieces obey a high-level musical structure which is not present in the melodies performed by participants. The question may be raised, what is the influence of this structure on style attribution? It is possible to set the scene for answering this question with the data obtained in the experiment.

To this aim, the data has been analyzed in terms of Signal Detection Theory by first calculating mean hit (or true positive) rates and mean false alarm (or false positive) rates, and then by calculating mean discriminability and mean response bias. This approach allows to separate two distinct factors: (a) a perceptual discrimination component and (b) a response bias, i.e., a tendency to answer *no* to all trials. We use the non-parametric measures *A'* for discriminability and *B”d* for response bias (Donaldson, 1992).

**Figure 8:**
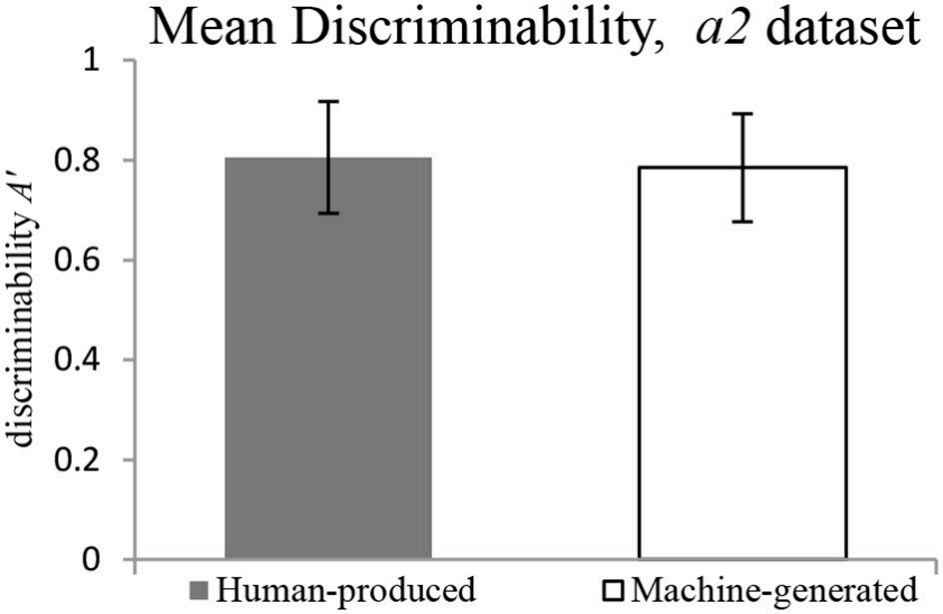
**Mean Discriminability for *a2* dataset**.

**Figure 9:**
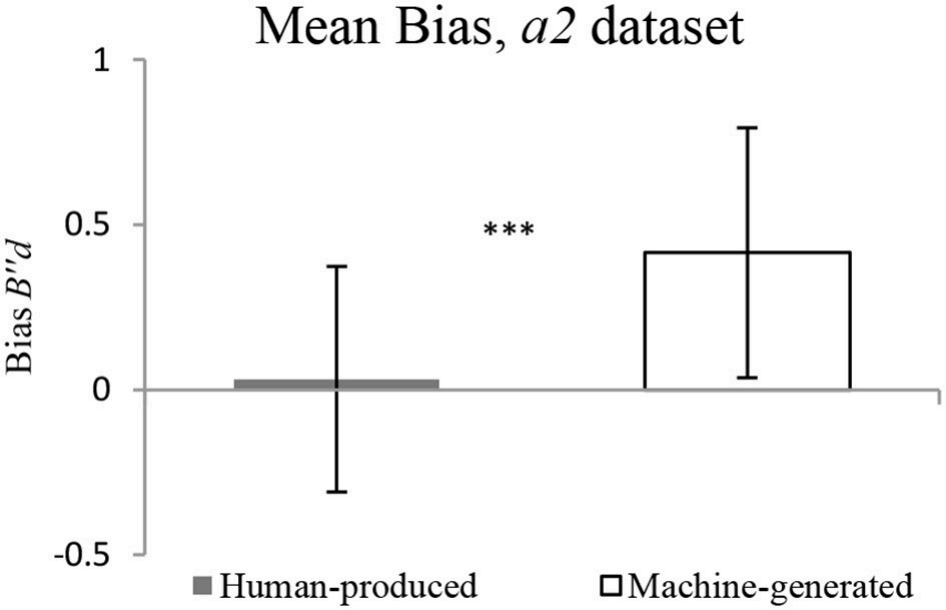
**Mean Response Bias for *a2* dataset**.

The findings on the *a2* dataset show that there is no significant difference in discriminability between *Human-produced* and *Machine-generated* conditions, as illustrated in Figure 8. The response bias is significantly more conservative for pieces: the participants have a tendency to respond *no* to the question “Does it sound like your music?” in the *Machine-generated* condition, as illustrated in Figure 9.

It must be noted that this holds true for the two cases of datasets, *a1* (original dataset) and *a2* (explainable errors dropped). The discriminability is revealed to be not significantly different for *Human-produced* and *Machine-generated* conditions, while the response bias is significantly higher in the *Machine-generated* condition. Table 1 gives details for different datasets.

**Table 1:** **Comparing *Discriminability* and *Response Bias***.

	***p*-values for Discriminability, *Human-produced* vs. *Machine-generated***	***p*-values for Response Bias, *Human-produced* vs. *Machine-generated***
*a1*	0.22	0.002
*a2*	0.30	0.001

p-values for Wilcoxon exact signed ranks test with Pratt zeros handling; a1 corresponds to the initial dataset, a2 corresponds to the dataset with explainable errors dropped.

This difference in response bias cannot be attributed to the specific sequence in which the experiment was conducted. Indeed, it could be hypothesized that the response bias might be higher at the end of the test phase (that is, in *Machine-generated* condition), due to the children's diminishing concentration for example. However, an additional analysis conducted separately on the first half and on the second half of *Machine-generated* condition, shows that there is no significant difference in the response bias. The mean response bias is of 0.5 and 0.3 for the first and second half respectively, but the difference is not statistically significant with a Wilcoxon test (*p* = 0.12).

While standard deviation of response bias is high, inter-individual differences do not seem to give a plausible explanation of this difference in response bias. There is a weak positive correlation (Spearman's rho = 0.31, *p* = 0.02) between the discriminability in *Human-produced* and *Machine-generated* conditions (*a2* dataset), which could suggest that participants who are good at recognizing their own melodies are good at recognizing pieces as well. However, no significant correlation was found for the response bias (Spearman's rho = 0.23, *p* = 0.09), suggesting that the response bias is not due to inter-individual differences.

Our finding on significant response bias is different from Flach et al. (2004) discussed in the section Human Performance in Recognition of Action Identity: in this study, no significant response bias was found, either for the original recordings of clapping or for altered recordings.

The difference between response bias in *Human-produced* and *Machine-generated* conditions could be interpreted in the following way. When these pieces were presented to the participants, to some extent they sounded new to them: while being composed on the basis of participant's action style, these pieces both (a) conformed to a software determined musical structure not present in the original training sets (as described in the sub-section Materials) and (b) had not been heard before by the participant. This could lead to a conservative response bias in answering to the question “Does it sound like your music?” in the *Machine-generated* condition.

This interpretation is partially supported by studies on music recognition as a function of mere exposure, which compare familiar and unfamiliar musical excerpts. At least to some extent, a parallel could be drawn between the melodies played and heard by the participant and the familiar excerpts on the one hand; and between machine-composed pieces and unfamiliar excerpts on the other hand. For example, Peretz et al. (1998) have reported that, in a recognition task, the response bias is significantly higher for new/unfamiliar melodies as compared to old/familiar ones (i.e., tendency to answer new). However, the authors also found evidence that the discriminability was better for old/familiar melodies than for new/unfamiliar ones, which is not the case in our study. This may mark a limit to a straight-forward comparison between classical studies on recognition memory and the recognition of one's own production and one's own style.

Dissociation studies are needed to elucidate the interactions between different types of memory effects, style recognition, and high-level musical structure, for instance, by controlling the structure of machine generated pieces as independent variable.

## Conclusion

In this study, we evaluated to what extent musically naïve school-age children were able to correctly attribute human-generated and machine-generated musical excerpts. The results show that the style attribution is well-above the chance level, and that the accuracy for machine-generated excerpts is practically equivalent to the accuracy for human-generated excerpts.

Moreover, these findings lead us to claim that the musical style of participants was well-simulated by the software and that the tested system, seen as an exemplar of many style simulation systems based on similar technologies, can be used in many situations where style recognition is crucial. This is the case for performance situations involving machine-learning software, which are increasingly prevalent in computer music.

Further studies that involve manipulating the Markovian constraints are needed to determine exactly which cues are used for self-recognition. However, the findings of this research may already have interesting applications, as an aid to composition or educational tools. Indeed, these tools confront the user with his or her own style replicated by computational processes, in such a way as to introduce new, stylistically consistent elements and to potentially enhance the perimeter of the user's future actions.

## Author contributions

The experiment was designed by AK and FP, the software was designed by FP, the experiment was conducted by VR and analyzed by AK.

### Conflict of interest statement

The authors declare that the research was conducted in the absence of any commercial or financial relationships that could be construed as a potential conflict of interest.

## Supplementary material

The Supplementary Material includes audio samples for Figures 3, 4, and 5.

Figure 3: Audio 1 for Subject's melody 1, Audio 3 for Subject's melody 2, Audio 2 for Subject's piece, Audio 4 for Other's piece.

Figure 4: Audio 5 for Subject's melody, Audio 6 for Other's melody.

Figure 5: Audio 7 for Subject's melody, Audio 8 for Other's melody, Audio 9 for Other's piece.
